# Histone Deacetylase Inhibition Downregulates Collagen 3A1 in Fibrotic Lung Fibroblasts

**DOI:** 10.3390/ijms141019605

**Published:** 2013-09-27

**Authors:** Xiangyu Zhang, Hui Liu, Thomas Hock, Victor J. Thannickal, Yan Y. Sanders

**Affiliations:** Division of Pulmonary, Allergy and Critical Care Medicine, Department of Medicine, University of Alabama at Birmingham, Birmingham, AL 35294, USA; E-Mails: xiangyuzhang70@gmail.com (X.Z.); huiliu@uab.edu (H.L.); thock@uab.edu (T.H.); vjthan@uab.edu (V.J.T.)

**Keywords:** histone deacetylase inhibitor (HDACi), suberoylanilide hydroxamic acid (SAHA), histone modification, epigenetic, collagen III, COL3A1, fibroblasts, idiopathic pulmonary fibrosis (IPF)

## Abstract

Idiopathic pulmonary fibrosis (IPF) is a deadly disease characterized by chronic inflammation and excessive collagen accumulation in the lung. Myofibroblasts are the primary collagen-producing cells in pulmonary fibrosis. Histone deacetylase inhibitor (HDACi) can affect gene expression, and some, such as suberoylanilide hydroxamic acid (SAHA), are US FDA approved for cancer treatment. In this study, we investigated SAHA’s effects on the expression of collagen III alpha 1 (COL3A1) in primary human IPF fibroblasts and in a murine model of pulmonary fibrosis. We observed that increased COL3A1 expression in IPF fibroblasts can be substantially reduced by SAHA treatment at the level of transcription as detected by RT-PCR; collagen III protein level was also reduced, as detected by Western blots and immunofluorescence. The deacetylation inhibitor effect of SAHA was verified by observing higher acetylation levels of both histone H3 and H4 in treated IPF cells. Chromatin immunoprecipitation (ChIP) experiments demonstrated that the reduced expression of COL3A1 by SAHA is with increased association of the repressive chromatin marker, H3K27Me3, and decreased association of the active chromatin marker, H3K9Ac. In our murine model of bleomycin-induced pulmonary fibrosis, the SAHA treated group demonstrated significantly less collagen III, as detected by immunohistochemistry. Our data indicate that the HDACi SAHA alters the chromatin associated with COL3A1, resulting in its decreased expression.

## 1. Introduction

Idiopathic pulmonary fibrosis (IPF) is a fatal disease with no effective treatment and an unclear pathogenesis [1]. It is characterized by distorted pulmonary structure accompanied by excessive deposition of extracellular matrix (ECM) proteins, such as collagen, and the presence of apoptosis-resistant myofibroblasts [2]. Collagen is one of the major constituents of lung connective tissue. There are different types of collagen. Collagen type I and III predominate within healthy and fibrotic lungs [3]. In IPF, both type I and III collagen production are increased [4]. Though the mechanism of excessive collagen deposition at fibrotic lesions is not clear, the myofibroblasts are a key cell type that is thought to be responsible for overproducing ECM proteins [5].

Studies to reduce collagen deposition and accumulation are important for the therapeutic control of lung fibrosis. A number of therapeutic strategies, such as anti-fibrotic agents and anti-cytokine therapies, have been examined without promising results [6]. To explore new drugs for IPF, we investigated the therapeutic potential of the histone deacetylase inhibitor (HDACi), suberoylanilide hydroxamic acid (SAHA, Vorinostat, Merck). SAHA is a US FDA approved drug for the treatment of cutaneous T-cell lymphoma [7].

Histone acetylation is balanced by two classes of enzymes, histone acetyltransferases and histone deacetylases (HDACs). HDACs function by removing acetyl groups on specific histones, resulting in chromatin conformational changes that reduce the ability of DNA to be transcribed [8]. HDAC inhibitors are chemicals that inhibit HDAC enzymes, which lead to increased transcriptional activity and upregulation of specific genes. If the upregulated genes are transcription factors, then they, in turn, can alter the expression of a multitude of genes, including downregulation of specific targeted genes. We previously reported [9] that SAHA can induce apoptosis in cultured human IPF fibroblasts. Others demonstrated that SAHA can abrogate the TGF-β effect on increasing collagen I deposition [10]; another HDAC inhibitor, trichostatin A, can inhibit collagen I mRNA induction by TGF-β in human lung fibroblasts [11]. However, the effects of SAHA on the increased collagen type III in IPF [12,13], as well as the associated changes of histone modifications with this gene (*COL3A1*) have not been explored.

In this study, we investigated if COL3A1 can be downregulated by SAHA in primary IPF fibroblasts and if its expression regulation is associated with histone modification alterations. We also examined if collagen III production can be inhibited by the SAHA treatment in a murine model of bleomycin-induced pulmonary fibrosis.

## 2. Results and Discussion

### 2.1. Increased COL3A1 mRNA in IPF Lung Tissues and IPF Primary Fibroblasts

**Figure 1:**
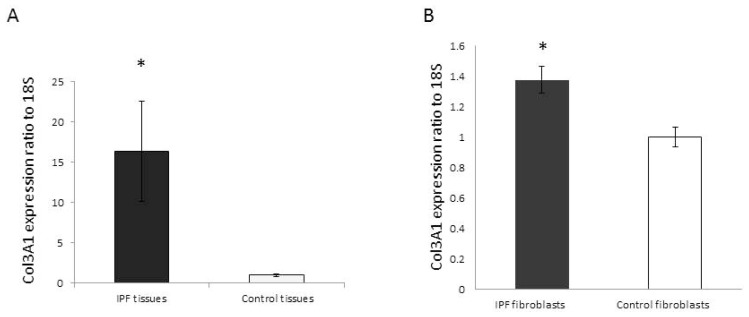
Idiopathic pulmonary fibrosis (IPF) lung tissues (**A**) and primary fibroblasts (**B**) have higher COL3A1 mRNA expression when compared to control samples by real-time RT-PCR. Results are the average of at least three independent experiments (IPF or Control lung tissue samples, *n* = 3). Bars indicate the mean ± SD. * *p* < 0.05 compared to the non-IPF control.

We first compared the COL3A1 mRNA expression in IPF and normal control lung tissues. Using real-time RT-PCR, significantly increased COL3A1 expression was detected in IPF lung tissues compared to control samples (Figure 1A). In cultured primary IPF, fibroblasts displayed moderately, but statistically higher COL3A1 expression (Figure 1B) compared to the control cells. These data are consistent with previously published microarray data [12,14].

### 2.2. SAHA Downregulates COL3A1 Expression

**Figure 2:**
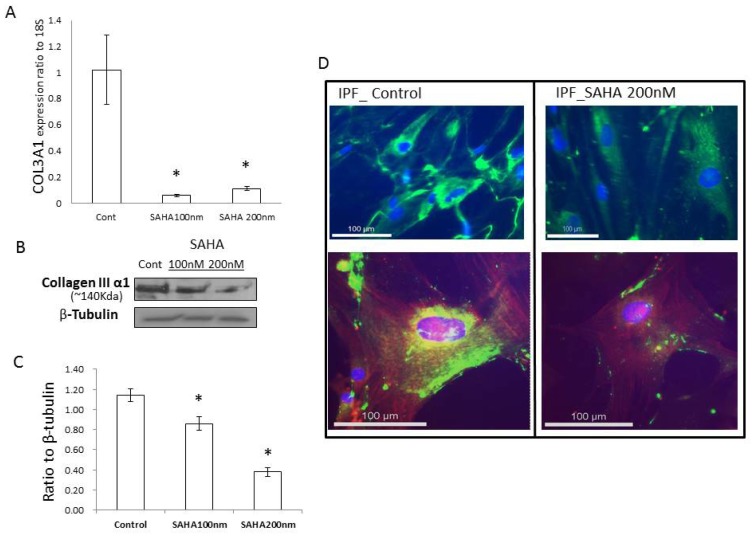
COL3A1 expression in IPF primary fibroblasts with or without suberoylanilide hydroxamic acid (SAHA) treatment. (**A**) Expression of mRNA COL3A1 in IPF cells with or without SAHA by real-time RT-PCR; (**B** and **C**) Expression of collagen III in IPF whole cell lysate, which indicates intracellular collagen III, by Western blot with 8% SDS-PAGE gel under reducing condition (more information is available in the online supplementary data). Collagen III α1 is at approximately 140 kDa marker [15]. β-tubulin was used as a loading control; **C** is densitometry by blots like **B**; (**D**) Representative pictures of immunofluorescence of IPF cells without (left) and with (right) SAHA, stained with α-SMA (red, bottom) and collagen III (green). Top panels: IPF fibroblasts stained with Col3A1 anti-body (green) indicating intracellular collagen III; nuclei are stained blue (DAPI). Bottom panel: IPF fibroblasts stained with Col3A1 (green) and α-SMA (red); nuclei stained blue (DAPI). The scale bar as shown at the left bottom corner is for 100 μm. The results are the average of at least three independent experiments. The bars indicate the mean ± SD. * *p* < 0.05 compared to the control.

Next, we examined if SAHA can downregulate COL3A1 expression in primary IPF myofibroblasts. Treating IPF myofibroblasts with 100 nM or 200 nM SAHA for 48 h resulted in significantly lower level of COL3A1 mRNA (Figure 2A). Western blots of the whole cell lysate, which mainly indicates intracellular collagen III, displayed lower collagen III under SAHA treatment (Figure 2B,C). We also examined the expression of α-smooth muscle actin (α-SMA), a characteristic marker of lung myofibroblasts. Western blotting showed that the upregulated α-SMA in these cells was also downregulated by SAHA (data not shown), as we and others showed in previous studies [9,10]. Immunofluorescent studies demonstrated less and weak intracellular collagen III staining (Figure 2D, green); less α-SMA fiber (Figure 2D, red) was also noticed after treating the IPF cells for 48 h with 200 nM SAHA. These data demonstrated that SAHA can downregulate COL3A1 at transcriptional and translational levels.

### 2.3. Histone Modifications Mediate the Downregulation of COL3A1 Expression

**Figure 3:**
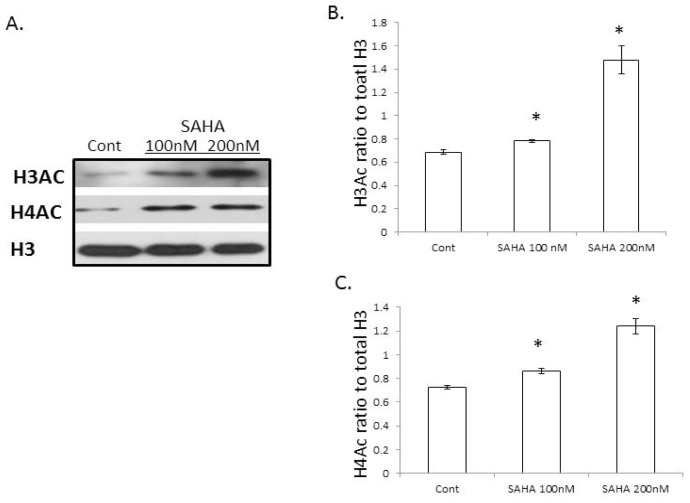
Acetylation of histone H3 and H4 in IPF fibroblasts with or without SAHA treatment. (**A**) Western blots of total acetylated histone H3 and H4 in IPF fibroblasts without or with SAHA treatment for 48 h. Total H3 was used as a loading control; (**B**) and (**C**) are densitometric analyses of Western blots, like **A**. The results are the average of at least three independent experiments. The bars indicate the mean ± SD. * *p* < 0.05 compared to the control.

**Figure 4:**
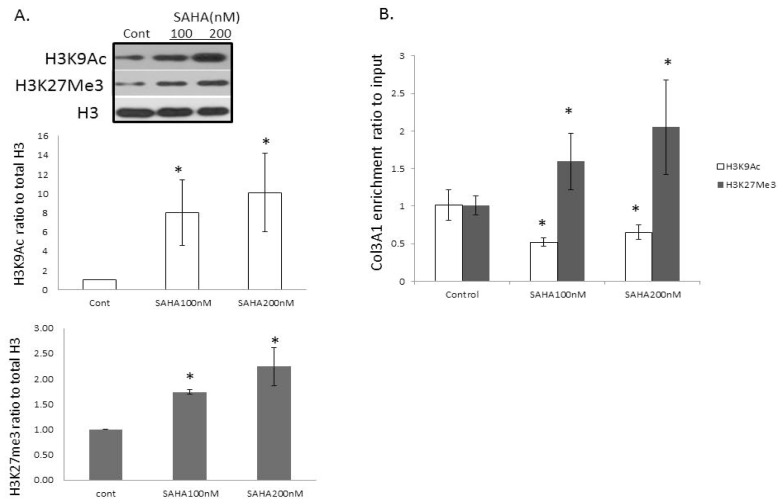
Other histone modification changes and chromatin immunoprecipitation (ChIP) assays demonstrated histone-association changes of COL3A1 with or without SAHA treatment in IPF fibroblasts. (**A**) Western blots of H3K9Ac and H3K27Me3 in IPF primary fibroblasts treated with or without SAHA for 48 h. Total H3 was used as a loading control. Bottom: densitometric analysis of blots, like **A**; (**B**) Association changes of histone modifications, H3K9Ac and H3K27Me3, with COL3A1 at the promoter region by ChIP assays. The quantitative ChIP assays were performed to examine the association changes of histone modifications, H3K9Ac (white bars) and H3K27Me3 (black bars), with the promoter region of COL3A1. The bars represent the relative levels of the PCR product of the region of COL3A1 associated with these specific histone modifications under the indicated conditions after immunoprecipitation with these specific antibodies, normalized to input DNA. The bars represent the mean ± SD from the average of at least three independent experiments. * *p* < 0.05 *vs*. the untreated control to its own group.

As SAHA is a histone modifier, we examined the changes in histones that are related to COL3A1. SAHA is a HDAC inhibitor; concomitantly, we observed that the total acetylated histone H3 and H4 are increased after treatment with SAHA (Figure 3). We then examined if there are other histone modifications, as well as association changes of histone modifications with COL3A1 by ChIP assays using specific histone modification antibodies. Figure 4A demonstrated that besides the histone acetylation changes, other histone modifications, such as the trimethylation of histone H3K27 (H3K27Me3), were also changed, in that H3K27Me3 is increased with SAHA treatment. ChIP assays (Figure 4B) showed a significantly increased association of the COL3A1 DNA sequence with the repressive marker, H3K27Me3, while the association with the active histone marker, H3K9Ac, is decreased. These changes were consistent with COL3A1 decreased expression level with SAHA treatment. These data indicate that the HDACi SAHA not only increases the acetylation of histone, but alters other histone modifications, as well as histone associations with specific genes, such as *COL3A1*.

### 2.4. SAHA Treatment Results in Decreased Collagen III Protein in a Murine Model of Bleomycin-Induced Pulmonary Fibrosis

**Figure 5:**
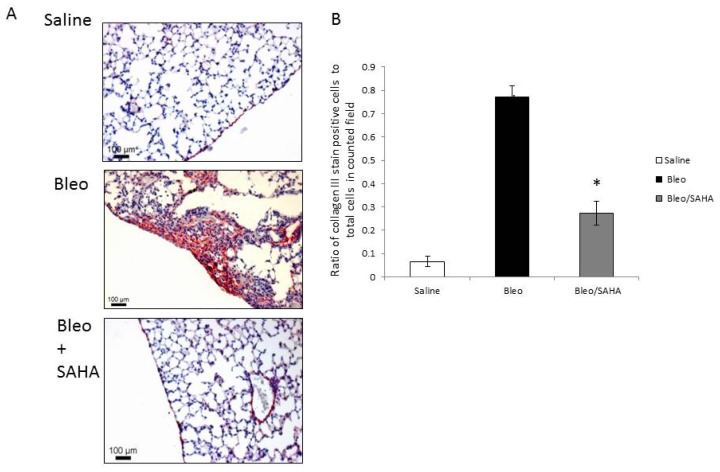
Collagen III in the murine model of bleomycin-induced pulmonary fibrosis with or without SAHA treatment. (**A**) Immunohistochemistry with antibody against collagen III in mice lung with saline, bleomycin and bleomycin with SAHA. Representative collagen III stained 5-μm sections of formalin-fixed, paraffin-embedded lung of six- to eight-week-old C57BL mice 28 days after intra-tracheal injection of normal saline (top), bleomycin (Bleo, middle) or bleomycin with SAHA (Bleo + SAHA, bottom) treatment every other day from day 10 to day 28 after bleomycin injury. Immunostaining of collagen III (red) is seen in large areas in the bleomycin only group. Comparatively, there is much less immunostaining of collagen III in the Bleo/SAHA treated group (bottom) compared to the bleomycin group, while little staining is observed in the saline group (a higher magnification of the figures is in the online supplementary data); (**B**) Quantitation of collagen III stained cells in randomly chosen fields relative to the total cell count in the field. The bars represent the mean ± SD of the average of at least three randomly chosen fields. * *p* < 0.05 *vs.* the bleomycin group.

Lastly, we examined collagen III in a murine pulmonary fibrosis model induced by bleomycin. One group was given SAHA every other day starting at day 10 after bleomycin injury when lung fibrosis develops. The vehicle of SAHA, HOP-β-CD, is a widely used nontoxic drug carrier that is well tolerated in mice [16]. All the mice were sacrificed on day 28 after bleomycin injury. Immunohistochemistry staining with a collagen III antibody demonstrated better lung structure and significantly less collagen III in the SAHA treated bleomycin group compared to the bleomycin only group (Figure 5).

In this study, we demonstrated that SAHA can downregulate type III collagen expression on transcriptional and translational levels; the downregulation is associated with specific histone modifications. We and others have also shown that HDACi can inhibit α-SMA and collagen synthesis [9,10]. There are studies showing that HDAC4 is important in TGF-β-mediated pathways that upregulate α-SMA and type I collagen [11]. SAHA is a broad spectrum HDAC inhibitor; it can inhibit various HDACs, including HDAC4. We do not know if the inhibition of collagen type III by SAHA is also due to the inhibition of HDAC4. However, we demonstrated that decreased association with the active histone marker, H3K9Ac, and increased association with the repressive histone, H3K27Me3, correlated with COL3A1 downregulation.

Increased gene expression of collagen III was observed in IPF tissues and in IPF primary fibroblasts. As SAHA is a histone modifier, we investigated the histone modification changes and associations with this gene. The association of H3K27Me3 with COL3A1 is in line with a report of COL3A1 expression in aging rat kidney, which demonstrated that COL3A1 has increased association with H3K27Me3 and reduced association with H3K9Me3 when compared to young rat kidney, yet H3K27Me3 and H3K9Me3 are both repressive histone markers [17]. However, in their study, COL3A1 is upregulated in the aging rat kidney; they reasoned, according to other studies [18], that it is possible by losing H3K9Me3 at the COL3A1 locus could allow increased activation of gene transcription despite the persistence of the repressive marker, H3K27Me3. We also examined the association of COL3A1 with H3K9Me3, but we did not detect an association with COL3A1 with SAHA treatment (data not shown). Nonetheless, in our model, the downregulation of COL3A1 by SAHA is likely achieved by the increased association of repressive histone marker, H3K27Me3, and decreased association with active marker, H3K9Ac. In this study, we did not examine the changes of histone-related enzymes, as one kind of histone modification may alter/affect other histone modifications, until a state of new balance is achieved [19]. SAHA may cause a new balance in various histone modification changes, with related histone enzymatic changes. Our data in this and another related study (data not shown, manuscript in submission) demonstrate that SAHA, although an HDAC inhibitor, not only increases histone acetylation, but also can affect other histone modifications, such as histone methylation, as well as the association of histone modifications with specific genes to alter gene expression.

SAHA is a broad spectrum HDAC inhibitor. It also has some other important non-histone substrates. One such substrate is p53, which can increase its acetylation [20] and, then, affect a wide range of gene expression. SAHA can also control cell cycle [21]. p21 has been reported as being able to be upregulated in cell culture with SAHA [22,23]. There are other studies demonstrating the downregulation of collagen in cells by various HDAC inhibitors [10,11], exploring mechanisms other than histone modification-associated changes. Here, we mainly explored the changes associated with histones, showing that related histone modification alterations are associated with changes in gene expression levels. However, we cannot determine if these histone changes are a direct effect from SAHA on histone modifications, or is a cause from SAHA by changing the expression of other genes that regulate the expression of collagen 3A1. There are reports that Trichostatin A, an HDACi from the same family as SAHA, can significantly enhance an NF-κB-dependent transcription [24]. These all could affect various transcription factors and/or gene expression, which then affect COL3A1 expression. SAHA may regulate gene expression in various ways. Since SAHA can downregulate the myofibroblast marker, α-SMA, there has been studies suggesting that it may inhibit the transition of fibroblasts to myofibroblast [10]. SAHA may not only inhibit the differentiation and proliferation [25] of the fibroblasts, it may also induce apoptosis of the myofibroblasts [26,27] and affect other non-histone substrates for gene regulation [20,21]. In this study, we used IPF primary fibroblasts, which usually have higher α-SMA and produce more collagen as myofibroblasts. We demonstrated that the intracellular collagen III, likely the newly synthesized collagen III, can be inhibited by SAHA after treating the cells for 48 h. However, we should point out that the collagen III shown by whole cell lysate is not an indication of matrix formation, which needs ascorbic acid for the ECM formation in cell culture. The intracellular collagen could have post-translational deficiencies, which would affect the extracellular matrix formation and may affect further gene expression by feedback mechanisms [28].

In the murine model of bleomycin-induced lung fibrosis, the mice group with SAHA treatment showed markedly less collagen III and better lung structure in the lung tissues when compared to the untreated control, which is probably not only due to the association changes of COL3A1 with specific histone modifications, but also the overall effects of SAHA.

Increased collagen deposition in the matrix is one of the major characteristics of IPF. Decreased collagen production is important in treating IPF. SAHA may not only affect gene expression by inhibiting histone deacetylases to increase histone acetylation, it may also affect other histone modifications, as well as histone association with specific genes. No matter how, our data support the concept that SAHA deserves further investigation as a potential drug for IPF treatments.

## 3. Experimental Section

### 3.1. IPF Lung Tissue Samples, Cell Culture and Treatment

This study was approved by the University of Alabama at Birmingham (UAB, Birmingham, AL, USA) Institutional Review Board. Lung tissue samples were obtained from UAB Tissue Procurement Facility of 3 de-identified IPF patients (mostly severe late stage) and 3 controls (failed donor, no known lung diseases). Human primary IPF fibroblasts were generous gifts from Dr. Carol Feghali-Bostwick (University of Pittsburgh) or from the UAB tissue procurement facility. The cells were kept in 10% fetal bovine serum (FBS) and Dulbecco’s modified Eagle medium (Invitrogen), with 1% penicillin/streptomycin. The cells were seeded at a density of 3 × 10^4^ cells/well in 6-well plates or as indicated. When cells were near 80% confluence, the culture medium was changed to 1% FBS medium and left overnight. Then, the cells were treated with DMSO only, as the control, or SAHA (Sigma Aldrich, St. Louis, MO, USA) at 100 nM or 200 nM for 24 or 48 h.

### 3.2. RNA Extraction and Quantitative Real-Time RT-PCR

RNA was extracted by an RNAeasy Kit from Qiagen (Valencia, CA, USA). One microgram of RNA was reverse transcribed into cDNA using a cDNA synthesis kit (Clontech, Mountain View, CA, USA). Real-time RT-PCR of COL3A1 was performed in triplicate and normalized to 18S with the ΔΔ*C*t method, as previously described [29]. The primers used in the PCR are listed in Table 1.

### 3.3. Protein, Nuclear Extraction and Immunoblotting

Protein from whole cell lysate was collected by 2× SDS-reducing sample buffer containing protease inhibitors. Nuclei were extracted with an Epiquick Nuclear extraction kit (Epigentek, Brooklyn, NY, USA).

Collagen III antibody (Col3A1 antibody catalog#49-394) was from ProSci Incorporated (Poway, CA, USA); anti-H3Ac (#9671), -H4Ac (#2591), -H4 (#2591) and -β-tubulin (#2128) were from Cell Signaling (Beverly, MA, USA). β-tubulin was used as the loading control for the whole cell lysate; H3 was used as the loading control for nuclear extracts. Western blots were carried out as previously reported [30].

### 3.4. Immunofluorescence Staining

Cells were cultured on cover-slips with or without SAHA for 48 h. Anti-α-SMA was from Biocarta US (San Diego, CA, USA). Antibodies of Col3A1 (1:400 dilution) and α-SMA (1:400 dilution) were used, with FITC- or Alexa-Flour 594 (Jackson Immuno Research, West Grove, PA, USA) conjugated secondary antibody at 1:200, respectively. The slides were examined with a Zeiss Axiovert 200M fluorescence/phase microscope with Axiovision LE software (Carl Zeiss International, Jena, Germany).

### 3.5. ChIP Assays

Chromatin immunoprecipitation (ChIP) assays were performed as per the manufacturer’s protocol (Epigentek, Brooklyn, NY, USA) with minor modifications [31]. Anti-H3K27Me3 (ab6002) and anti-H3K9Ac (ab10812) were from Abcam (Cambridge, MA, USA). ChIP-DNA was amplified by real-time quantitative PCR with the primers listed in Table 1. All results were normalized to input DNA.

### 3.6. Animal Model and Immunohistochemistry

The animal studies were performed in accordance with the University of Alabama at Birmingham Institutional Animal Care and Use Committee approved protocols. Six- to eight-week C57BL mice were used. A single dose of normal saline or bleomycin sulfate at 3U/kg body weight was instilled intratracheally. SAHA in HOP-β-CD solution [32] was fed by mouth every other day at 20 mg/kg, starting day 10 after bleomycin injury. Mice were sacrificed on day 28 after the injury. The lungs were prepared for histology studies by fixed in formalin for 24 h, then 70% ethanol, before being sent for paraffin embedment. Antigen retrieval was performed on paraffin-embedded sections by heating in pH 6.0 citrate buffer for 20 min. The primary antibody of collagen III was used at 1:600 dilutions. Staining was developed with biotinylated anti-rabbit secondary antibodies; after washing, alkaline phosphatase-conjugated streptavidin was added. Color development was performed using vector red AP substrate (Vector Labs, Burlingame, CA, USA). Slides were counterstained with hematoxylin QS. Images were obtained with a Nikon TE2000U microscope equipped with a QiCam Fast Cooled high-resolution CCD camera with MetaMorph software (v.6.2r4, Universal Imaging, West Chester, PA, USA). At least 3 random fields were counted for COL3A1 stained cells and then ratio to all the cells in the field.

### 3.7. Statistical Analysis

Data are expressed as the mean ± standard deviation (SD). The Student’s *t-*test or one-way ANOVA for comparisons involving three or more groups were performed. A *p*-value of 0.05 or less was used to determine statistical significance.

## 4. Conclusions

SAHA can downregulate the expression of collagen III in IPF primary fibroblasts and in a murine model of pulmonary fibrosis. The downregulation of collagen III in the IPF fibroblasts is associated with specific histone modifications. Understanding the mechanism of this regulation can be important for future drug development for IPF.

## Figures and Tables

**Table 1:** PCR primers used in this study.

	Gene Name	Gene ID	Sequence
RT-PCR	*Col3A1*	ENSG00000168542	F: 5′-ATTGCCTGGGATCACTGGAGCAC-3′
			R: 5′-CTGGTTTCCCACTTTCACCCTTG-3′
	*18S*	NR_003278	F: 5′-GTCTGCCCTATCAACTTTCG-3′
			R:5′-ATGTGGTAGCCGTTTCTCA-3′

ChIP PCR	*Col3A1*	ENSG00000168542	F: 5′-TGAAGGGCAGGGAACAACTTGATG-3′
			R:5′-ATGAAGCAGAGCGAGAAGTAGCCA-3′
